# The Development of White-Asian Categorization: Contributions from Skin Color and Other Physiognomic Cues

**DOI:** 10.1371/journal.pone.0158211

**Published:** 2016-06-29

**Authors:** Yarrow Dunham, Ron Dotsch, Amelia R. Clark, Elena V. Stepanova

**Affiliations:** 1 Department of Psychology, Yale University, New Haven, CT, United States of America; 2 Department of Psychology, Utrecht University, Utrecht, The Netherlands; 3 Department of Psychology, University of Southern Mississippi, Hattiesburg, MS, United States of America; Bournemouth University, UNITED KINGDOM

## Abstract

We examined the development of racial categorizations of faces spanning the European–East Asian (“White–Asian”) categorical continuum in children between the ages of four and nine as well as adults. We employed a stimulus set that independently varied skin color and other aspects of facial physiognomy, allowing the contribution of each to be assessed independently and in interaction with each other. Results demonstrated substantial development across this age range in children’s ability to draw on both sorts of cue, with over twice as much variance explained by stimulus variation in adults than children. Nonetheless, children were clearly sensitive to both skin color and other aspects of facial physiognomy, suggesting that understanding of the White-Asian category boundary develops in a somewhat different way than understanding of the White-Black category boundary, in which attention to features other than skin color appear only somewhat later. Discussion focuses on the implications of these findings for theories of social categorization.

## Introduction

Research on intergroup social cognition focuses on how and when individuals come to be thought of and treated differently as a function of the groups to which they belong. For example, individuals come to be thought of as clustering into racial, ethnic, national, and religious groups, and these classifications have implications for how members of those groups are perceived [1]. Thinking about, perceiving, or treating someone differently based on the groups to which they belong presupposes a prior process of categorization, i.e. of deciding who belongs to what groups in the first place [2,3]. That in turn requires identifiable perceptual cues, such as differences in physical appearance, language, dress or adornment. Much prior literature in this area has focused on this question via the Black-White racial distinction as it plays out in the U.S. context. This is understandable given the specific legacy of slavery and segregation in North America, but increasing diversity and recent patterns of migration necessitate a more inclusive approach. The present research therefore focuses on the perceptual differences that underlie the racial distinction between “White” and “Asian”, to be understood here as European / Caucasian on the one hand and East Asian on the other. This focus is valuable given the increasing number of Asian-American as well as multi- and bi-racial individuals with Asian heritage in the United States. This practical motivation is coupled with a conceptual one: it is not clear whether conclusions derived from the study of Black-White racial contrasts will generalize beyond that context. For example, other racial contrasts may differ in the relative salience of different morphological features such as skin color. Thus, it is critical to expand the empirical focus to other socially salient racial contrasts that better reflect the complexities of contemporary demography [4]. Here we approach this topic by asking how *skin color* and other *physiognomic features* jointly and independently influence categorization of a target face as White versus Asian, and how the influence of each of those features changes over early to middle childhood, ages over which which many social categories (including race and ethnicity] are rapidly growing in importance. This work thus contributes to our understanding of the development of race-based perceptual categorization for a less-studied racial contrast.

There is evidence that sensitivity to race category boundaries begins to appear quite early in development. For example, 3-month-old infants preferentially attend to faces of the same race as their primary caregivers [5,6], and 9-month-olds habituated to White or Asian faces recover from habituation when presented with a face of the other race [7], suggesting a basic form of perceptual categorization based on common features. Research with older children, primarily focusing on the White/Black racial distinction in the United States, suggests ceiling-level performance on simple categorization tasks by around age 7 [8–11]. However, there is also evidence that race-based categorization over these years is surprisingly fragile. For example, if more than two categories are included in a labeling task (e.g. having to correctly label White, Black, and Asian targets instead of just White and Black), performance plummets [12,13]. This raises the possibility that common forced-choice methodologies, as well as polysemy between color terms and category labels in the specific case of the Black-White contrast, could have inflated past estimates of categorization ability. In particular, it provides reason to think that children’s performance might differ dramatically when presented with a wider range of continuous and more complex variation, such as they must regularly encounter “in the wild”.

Against this backdrop, we recently investigated the Black/White perceptual distinction in North American adults and children between the ages of 4 and 9 [14], adapting a paradigm from adult-focused research [15] which dissociates the influence of skin color from other aspects of facial physiognomy. This is done by independently varying each of those two factors across 10 equally spaced levels and fully crossing them, creating a stimulus set of 100 faces in which skin color and other aspects of facial physiognomy are uncorrelated. We saw this as a first step towards understanding how children approach racial categorization when the stimuli exhibit a broader range of variation, and in particular how they make use of skin color versus other physiognomic factors. Replicating past research [15], adults were exquisitely sensitive to both factors, though in our data skin color was the more powerful cue. Children, however, showed a quite different pattern. Prior to the elementary school years, there was no evidence that children paid *any attention* to physiognomy, instead making judgments based solely on skin color. As they entered the school years, attention to physiognomy reliably increased, but at the end of the age range examined (age 9), attention to physiognomy remained weak, accounting for only a small fraction of the weight accorded to skin color. Thus, skin color dominated early race judgments with respect to the White-Black racial distinction.

However, skin color may play a more dominant role in Black-White racial categorization than it does in other racial distinctions. This could come about if, for example, skin color is simply the most salient visual cue in the input, such that it is seized upon and used as an initial heuristic for category identification. It could also be reinforced by the previously noted use of color terms to refer to the social categories themselves (i.e., “Black” and “White”). These issues do not necessarily arise for other racial contrasts. Further, the White-Asian contrast that is our focus here is, at least according to common intuition, less dramatically marked by skin color differences, though skin color clearly does contribute to categorization decisions [16], perhaps especially for children [17]. With these considerations in mind, we argue that a richer understanding of the perception and categorization of race requires moving beyond the usual focus on the White-Black distinction, which represents just one of many salient racial contrasts in contemporary North America and so should not be taken as the representative case of racial differentiation.

We thus explored the development of attention to skin color and physiognomy in 4-9-yr-old children and adults, employing the same task design we previously used to investigate White-Black racial categorization. For ease of exposition, we will reserve the term “physiognomy” for facial features *other than* skin color, though we acknowledge that skin color can be considered an aspect of racial physiognomy. We also note that “skin color” as we employ it here and as realized in our stimuli could incorporate influences from textural or reflectance properties that do not perfectly overlap with the notion of skin color as commonly used; this is generally true of experiments that cannot carefully control all aspects of the viewing experience across participants. In any case, in prior work focusing on the White-Black distinction, children’s judgments were dominated by attention to skin color, and even in adults skin color exerted approximately four times the influence of physiognomy. Because this sort of task had not yet been used to explore the White-Asian contrast in adults, the developmental end state was not wholly clear. However, based on past work described above, we expected adults to attend to both skin color and physiognomy in a balanced manner. Further, given that children’s judgment of the White-Black continuum were relatively insensitive to physiognomy, coupled with the intuition that skin color is a less salient guide to the White-Asian distinction, we suspected that White-Asian category judgments would be more difficult for children, leading to somewhat worse performance over all. That is, the nature of the White-Asian distinction might actually reflect a more demanding perceptual learning task, which would appear in the form of a more protracted learning period.

We now turn to a more focused set of competing hypotheses. A first possibility is that the constellations of physiognomic features that underlie racial differentiation are simply quite difficult for younger children, to the extent that they are only able to reliably extract them starting in the elementary school years. If so, we might expect younger children’s performance on the White-Asian distinction explored here to be quite bad, because it relies more heavily on precisely the perceptual dimensions that are most difficult for them. This would likely result in one of two patterns in the data. On the one hand, an inability to encode feature clusters that define the category boundary could result in noisier performance overall. On the other hand, an inability to encode those physiognomic features might lead children to attempt to map the category boundary to features they can more readily perceive, such as skin color differences; this might lead them to overweight skin color cues and perform reliably but quite differently from adults, who presumably attend to both kinds of cues. Evidence broadly consistent with the latter possibility comes from a study employing a match-to-sample face recognition paradigm with children and adults, in which children were overly reliant on pigmentation as compared to other physiognomic cues when making similarity judgments about faces [17].

However, other research on face processing in children provides some reason to suspect that children at least in principle have the perceptual acuity to identify the physiognomic cues underlying racial category boundaries. For example, children are sensitive to the subtle aspects of races underlying expressions of emotion [18] and judgments of warmth and competence [19]; they even show the “other race effect”, i.e. better recognition of members of their own racial group [20]. Given this, the powerful attention to skin color in the Black-White case might arise not because children *cannot* attend to other aspect of facial physiognomy but because skin color is such a salient and/or readily accessible cue that it serves as an initial heuristic that allows for reasonably successful initial category judgments. On this view, we might expect attention to physiognomy to make an earlier appearance with respect to the White-Asian category boundary, simply because skin color alone is not as diagnostic, leading children to identify and focus on a broader set of cues.

In summary, our investigation starts with the question of how skin color and physiognomic cues are weighted in adult White-Asian race category judgments. We then assess how children in the preschool to middle-elementary-school years make these judgments, asking whether employing physiognomic cues appear to be more difficult even for a category contrast that may more centrally rely on such cues. Answering these questions addresses a fundamental aspect of intergroup social categorization for a less-well-studied White-Asian race contrast which is also potentially a more challenging case study of perceptual differentiation. This is of interest for its own sake, but will also enable a more nuanced understanding of prior work that focused on the White-Black category boundary, especially with respect to whether children’s lack of attention to physiognomic cues in that instance reflects the general difficulty of such cues or rather something particular to that category distinction.

## Materials and Methods

### Ethics statement

The research described here was reviewed and approved by Yale University's Human Research Protection Program, FWA#00002571. Written consent was secured from adults and from parents of participants under 18; in addition, verbal assent was secured from participants under age 18. All measures, conditions, and data exclusions are fully reported. A portion of the data reported here was collected as part of an undergraduate thesis undertaken by the third author, A. Clark. Data is available at an online repository hosted by the Open Science Framework at osf.io/q5j8g.

### Participants

Past research focusing on the Black-White distinction exhibited appropriate power in a similar design with a sample of 76 children [13]; we therefore set our target sample at this same level, ultimately enrolling 75 children between the ages of 4 and 9 (*M* = 6.86 years, *SD* = 1.68 years, *range* = 4.0 to 9.92 years, male = 36). Children were recruited from a database of local children and from visitors to a science museum in New Haven, CT, USA. Most participants were White (78.6%), with smaller numbers of Asian (4%) and other/mixed race (17.3%) participants. Adult participants were recruited from a university-run participant pool, and were compensated with course credit. Data for two adult participants were dropped prior to analysis because those participants were determined to not meet study pool eligibility requirements. This resulted in a final sample of 112 adults aged 18 to 21 (male = 53). The adult sample was more racially diverse than the child sample (White = 50.4%, Asian = 26.1%, and other/mixed race = 23.4%). We also conducted a follow-up adult replication study with a different stimulus set (described below). This sample consisted of 100 adults (male = 53) recruited through Amazon’s Mechanical Turk; they were older (*M* = 34.8 years, *SD* = 11.1 years) and primarily White (81%; Black = 10%, Asian = 2%, Hispanic/Latino = 6%, other = 1%).

### Facial stimuli

Stimuli were developed using custom made Python scripts based on the FaceGen 3.5 SDK. FaceGen facial models are based on 3D laser scans of multiple adult faces, producing a high-dimensional computational model of face space (for other uses of this modeler in face perception research, see [21,22]. To create our stimuli, “base faces” were first produced by beginning with a prototypical “average” European male face and a prototypical “average” East Asian male face. To create several distinct sets of stimuli we introduced a small amount of random noise to each dimension, thereby producing base faces that remained highly prototypical for that racial category but differed slightly from FaceGen’s average face and from one another. Each of the resulting base face pairs were then used to create a unique grid of 100 faces by stepping from one face to the other independently over 10 steps of skin color and 10 steps of facial physiognomy (i.e., 10 levels of skin color from maximally White to maximally Asian were crossed with 10 levels of physiognomy, from maximally White to maximally Asian). In total we created three grids of 100 faces each, and adult participants were randomly assigned to one of these three grids.

**Fig 1:**
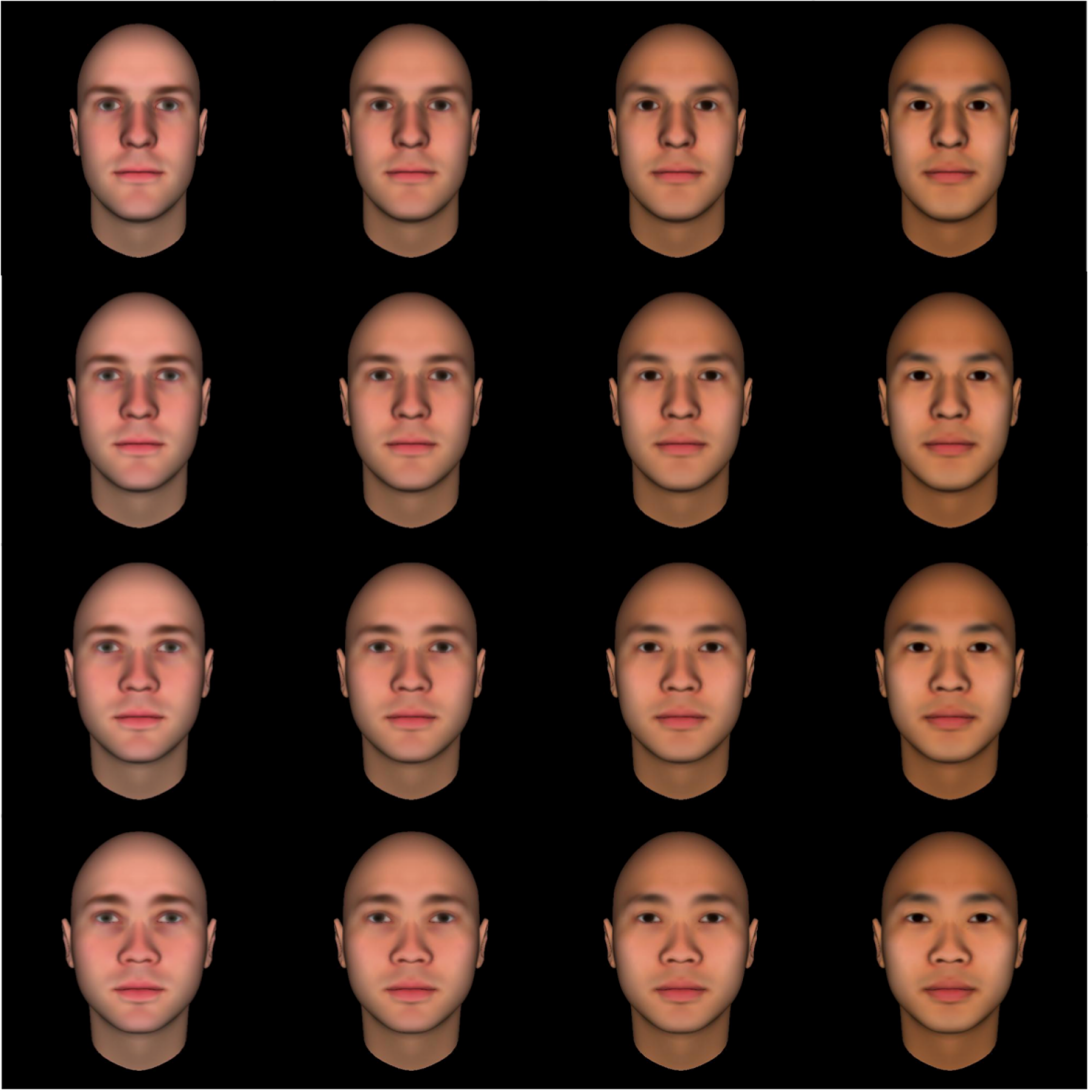
Example stimuli representing positions 1, 4, 7, and 10 on each dimension. Rows represent shifts in physiognomy; columns represent shifts in skin tone.

Because children could only complete a smaller number of trials and we wanted to ensure that each face was seen by a sufficient number of participants, all children viewed faces drawn from the first of these grids. In broad strokes, compared to the most prototypical European face, the most prototypical East Asian face had darker, more yellow-orange-tinted skin, lighter cheeks, lighter eye sockets, and more flushed lips; it also had higher brows, higher cheekbones, a larger forehead, a wider face, a shallower nose bridge, and a flatter nose. Example stimuli are presented in Fig 1.

**Fig 2:**
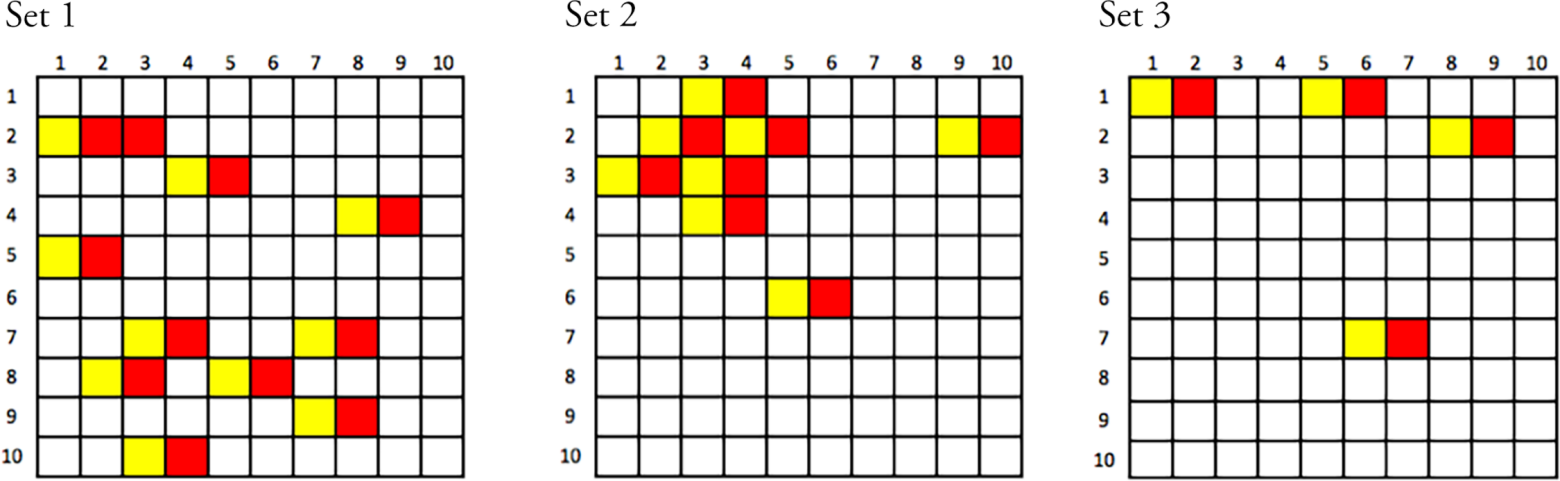
Summary of omissions in each stimulus set. Rows represent levels of physiognomy, columns represent levels of skin color / hue. Red fill indicates a missing cell; yellow fill indicates a stimulus that was duplicated.

Unfortunately, after data collection was complete, we identified an error in the code that converted FaceGen’s proprietary face space coordinate files to the standard image files used in the experiment. In brief, our code generated faces with the FaceGen SDK and then exported those faces as the image files that were used in the actual experiment. The generation and export process were imperfectly synced, such that the same face would occasionally be exported twice, resulting in two identical stimuli appearing in the set. When that occurred, the next face in sequence was omitted. That is, some cells in the 10 x 10 matrix of faces were omitted, and for every omission there was also a duplication. In total, across the three stimulus sets, 22 of 300 faces (7.3% of stimuli) were subject to this error, leading to 22 repeated faces and 22 correspondingly missing cells. These omissions appeared to be randomly distributed across the stimulus sets, and are depicted in Fig 2. Because different cells were omitted for each of the three stimulus set, all cells are represented in the adult data when collapsing across stimulus set. However, for the child data, which employed only one stimulus set, some cells are missing. While unfortunate, there are several reasons to believe that this issue does not seriously compromise the quality of our data. First, child participants received a random subset of 50 of 100 trials, meaning that many participants would not have categorized the missing stimuli even had they been present in the data. Second, all 10 levels of both predictors are represented in the data (though the missing cells mean they are not fully crossed in any one stimulus set). Third, as we present in more detail at the conclusion of the results section, below, we performed a series of supplemental analyses to determine whether responses were appropriately sensitive across the full range of variation despite the missing cells; these analyses did not raise any concerns. Fourth, we conducted a replication study with a different sample of adults using a new and complete stimulus set to further ensure that our data were not compromised by the missing stimuli.

### Face rating task

Adult participants viewed all 100 faces from one of three stimulus sets, or for the replication study (described below), 100 faces from a fourth stimulus set. To reduce the participant burden for younger children, child participants viewed a random subset of 50 faces drawn from the first stimulus set only. Faces were presented one at a time in the center of a computer screen. Participants placed each face on a linear continuum ranging from the most racially prototypical European face to the most racially prototypical East Asian face, constituting a 100-point scale. Anchor stimuli representing a racially prototypical face of each race were shown at either end of the linear scale. The anchor stimuli for each stimulus set were the racially prototypical faces from one of the stimulus sets not rated by the participant. The direction of the rating scale (i.e., White to Asian or Asian to White) was counterbalanced across participants.

### Procedure

The main study was conducted on a laptop computer. Child participants were tested by a White female experimenter either in a testing room at the lab (56% of children) or at a booth in a museum setting (44% of children). Children sat in front of the computer and the experimenter explained that they would play a game in which they would see many people and get to decide who was who. The experimenter advanced to a screen that showed two racially prototypical anchors, one European and one East Asian. In order to ensure that children understood that the task would involve racial categorization, it was explained that one of the faces is typically considered Asian or Asian-American, and the other anchor is typically considered White or European-American. The experimenter then advanced to a second screen showing the same two racially prototypical anchor faces positioned at either end of a linear scale. Children were told that on each screen, a face would appear in the center, and that their job would be to look at that face and decide where along the scale it belonged. They were then verbally instructed on use of the scale as a measure, ranging from a perfect match with one category through several intermediate possibilities to a perfect match with the other category. Because pilot testing revealed that some children appeared anchored to the extreme values, after instruction children categorized a practice stimulus that was intermediate along both the skin color and physiognomy dimension. If a child indicated that the stimulus was a perfect match for either of the racially prototypical anchors, they were reminded of the possibility of using the full range and then proceeded to the main task, in which 50 randomly selected target stimuli were presented one at a time at the center of the screen. Children pointed to the position on the scale where they wanted the marker to be placed, and the experimenter placed the marker accordingly. The experimenter would proceed to the next trial after assent from the child that they were satisfied with the marker placement. Children were offered a short break after 30 trials to encourage continued attention. The procedure for adults was similar but instructions were presented in writing, they saw all 100 faces constituting a full stimulus set, and they recorded their own responses on a slider. They were tested in a private testing room in a university research lab. Finally, the adult replication study was conducted online; participants completed the study on their own computer after being recruited from Amazon’s Mechanical Turk.

### Analysis

To respect the possibility of individual variation in attention to skin color and facial features, ratings were analyzed in a multilevel model with trials nested within participants and with random correlated slopes and intercepts, fit using Restricted Maximum Likelihood. Following Gelman and Hill [23], we report estimated regression coefficients and 95% confidence intervals around those estimates. The two primary coefficients (unstandardized betas) associated with the main predictors of skin color and facial features each represent the predicted effect on ratings of an equally spaced one-step shift in each respective dimension. Because the full range of ten steps amounts to the full range of variation between a highly prototypical White versus Asian face these coefficients can be compared to provide a relative sense of the extent to which each dimension affects judgments. However, this comparison must be interpreted carefully because if the total range of variation in one dimension is larger or more psychologically impactful than the other, a one-tenth step in that dimension would also be larger. That is, the fact that we partitioned each dimension into ten equal steps should not be taken to imply that the step-sized *across dimensions* have been equated. However, we are able to gain additional purchase on this issue by comparing the residual variance of explanatory models (i.e., models including predictors) with the residual variance of “unconstrained” models with random intercepts but no predictors, or with, for example, a model with only one of the two primary predictors; the proportional reduction in residual variance from an unconstrained to a nested explanatory model is an analog to variance-explained (*R*^*2*^) for the multi-level context [24,25], and therefore serves as an independent measure of the degree of explanatory power associated with a given predictor and the perceptual dimension it represents.

## Results

Because adult performance on this particular face-rating task was unknown, and because developmental findings should be interpreted in the light of mature performance, we begin our presentation with adults, then turn to children, and then to analyses that compare across age groups.

### Adult racial categorization

Preliminary inspection of adult response patterns from the main laboratory sample indicated three problematic participants, two who responded with the same value for all trials, and one who exhibited an inverted pattern such that all Asian physiognomy and skin color cues were responded to as if they were White cues, and vice versa. Data from these three adults were therefore dropped (attrition = 2.7%), leaving 109 usable participants; however, no substantive result reported here depends on these exclusions. No effects of participant gender appeared in the analyses described below.

#### Effects of stimulus set

An initial model predicting racial categorization as a function of skin color, physiognomy, and stimulus set accounted for 70.7% of the trial-level variance that was unexplained in the unconstrained model. The effects of skin color, physiognomy, and the interaction between them were significant, but were qualified by unexpected effects of stimulus set, both as main effects and interactions with the other predictors. Because the influence of our predictors of interest was qualified by these interactions, it is important to understand their influence. We therefore fit models with our question predictors (skin color, physiognomy, and their two-way interaction) separately for each stimulus set. Results are presented in Table 1, which depicts the 95% confidence intervals around the three parameters of interest for each stimulus set, as well as for the aggregated model including all three sets and a replication set, described further below. Directionally, all effects are consistent across stimulus sets, though there is some variation in magnitude. Most notably, the interaction between skin color and physiognomy is weaker in Set 3, its confidence interval just barely exceeding 0. However, the increased variance explained by including stimulus set and all its associated interaction terms in our main model is modest, representing less than 1% of additional residual variance, the same broad patterns hold across sets, and the maximally prototypical faces for each set were clearly judged as such (Table 1, last columns). For ease of interpretation we therefore proceed with a model collapsing across stimulus set, though we return to a discussion of stimulus differences and what might underlie them in the discussion.

**Table 1:** 95% confidence intervals describing estimate of each parameter of interest in adults as well as the mean value of the maximally prototypical Asian and White faces, separately for each of three stimulus sets as well as the primary model collapsing across stimulus set and a subsequent replication set. All estimates are in units on the categorization scale, ranging from 1 to 100.

	Intercept	Skin Color	Physiognomy	Skin x Phys	Asian	White
Set 1	[43.90; 47.04]	[6.51; 7.50]	[6.07; 6.95]	[.23; .35]	[90.2; 96.8]	[3.1; 8.5]
Set 2	[45.92; 49.36]	[5.68; 6.99]	[4.78; 5.95]	[.21; .33]	[88.0; 94.7]	[5.5; 11.6]
Set 3	[48.91; 52.75]	[5.06; 6.41]	[5.56; 6.75]	[.01; .13]	[91.3; 96.5]	[5.6; 12.2]
Sets 1–3	[46.91; 49.09]	[6.00; 6.73]	[5.72; 6.37]	[.17; .24]	[91.2; 94.8]	[6.0; 9.5]
Replication Set	[41.66; 44.67]	[6.75; 7.03]	[5.59; 5.89]	[.26; .36]	[87.5; 92.1]	[5.8; 9.4]

#### Primary analyses

Here we describe our main model, collapsing across sets and so including terms for skin color, physiognomy, and their two-way interaction. The intercept, representing the mean rating across all stimuli, was 48.00 [*CI* = 46.91; 49.09], very near the scale midpoint of 50. There were significant effects of both skin color, unstandardized beta (*B)* = 6.36 [*CI* = 6.00; 6.73] and physiognomy, *B* = 6.04 [*CI* = 5.72; 6.37], as well as an interaction between them, such that the effect of physiognomy was stronger when skin was darker, *B* = 0.21 [*CI* = 0.17; 0.24]. In addition to the similar size of the estimates associated with each dimension, calculation of the residual variance associated with each term in isolation further suggested they were contributing approximately equally, with 36.2% variance associated with skin color and 32.7% associated with Physiognomy.

**Fig 3:**
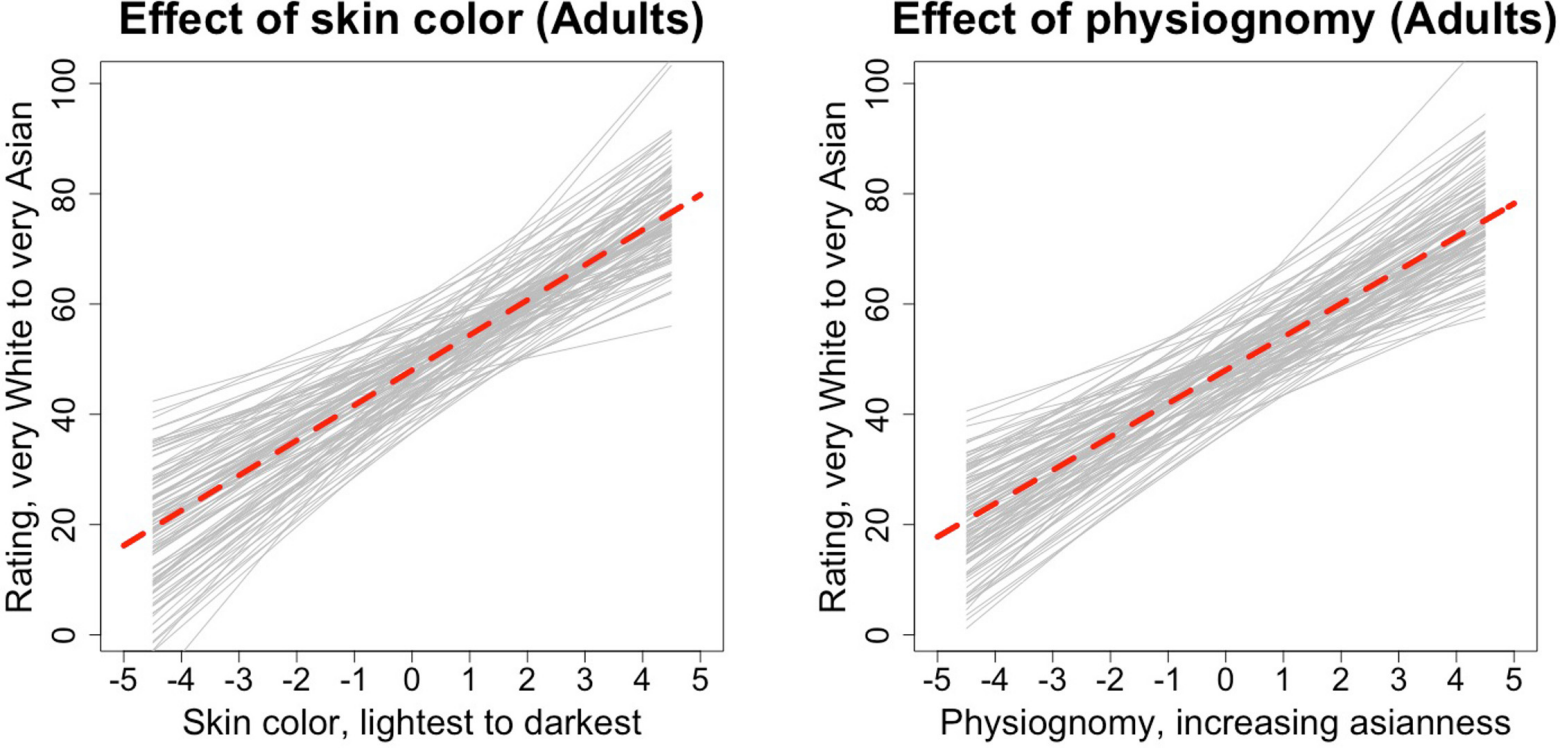
Main effects of (mean-centered) skin color and facial features when the other factor is set to its mean level, adult participants only. Dashed red line represents overall model predictions and light grey lines represent the predictions for each participant, estimated from the random effects component of the models.

**Fig 4:**
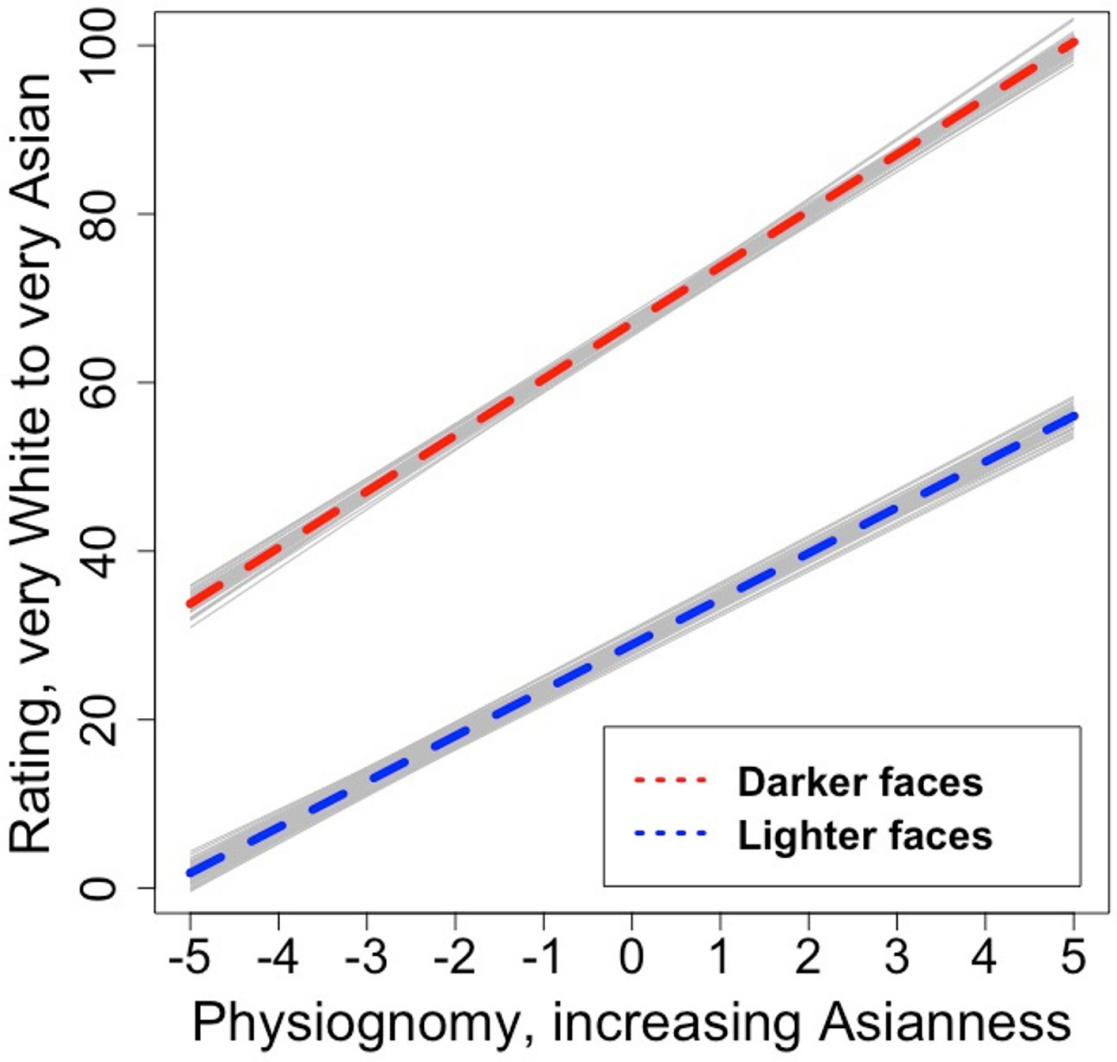
Interaction between facial features and skin color for adults. Lighter and darker faces are depicted at the 3^rd^ and 8^th^ level of skin color, respectively. Light lines indicate uncertainty in the fitted regression.

Fig 3 depicts the effect of each of skin color and physiognomy when the other factor is set to its mean value; Fig 4 depicts the interaction by plotting the effect of physiognomy for lighter and darker faces, defined as the 3^rd^ and 8^th^ level of skin color respectively. At these levels of skin color the two slopes differ, *B*_*skin = 3*_ *=* 5.48 [*CI* = 5.14; 5.82], *B*_*skin = 8*_ *=* 6.53 [*CI* = 6.19; 6.87]. However, this interaction, while highly significant, accounts for only a small portion of additional trial-level variance, (< 1%). It nonetheless indicates that the impact of physiognomy is larger for faces that are more Asian in color and hue. This effect is particularly interesting because in prior work focusing on the White/Black distinction [14,15], an interaction between skin color and physiognomy was also reported, but the direction of the effect was precisely the opposite: Less rather than more attention to physiognomy as faces were darker. We return to this issue in the discussion.

#### Effects of participant race

Our adult sample was approximately 50% White, but also included a substantial number of East Asian participants (26%). The remainder of the sample did not report race or identified with another racial group. To explore effects of participant race we therefore focused solely on White and Asian participants, fitting the same main model described above but with an additional dummy variable coding for participant race. Two effects of interest emerged from this analysis. First, Asian participants rated faces as somewhat less Asian overall, *B* = -4.17 [*CI* = -6.39; -1.96]. Second, while neither two-way interaction between participant race and skin color or physiognomy approached significance, there was a highly significant three-way interaction between skin color, physiognomy, and participant race, such that the interaction between skin color and physignomy described above was considerably stronger in Asian participants than White participants, *B* = .30 [*CI* = 0.21; 0.38], though it remained different from 0 in White participants, *B* = .11 [*CI* = 0.06; 0.16]. In simple terms, then, the effect of skin color was stronger for Asian participants when physiognomy was more Asian, and vice versa. However, like the other interaction terms described above, including the race term and its interactions led to only a small additional increase in trial-level variance explained, < 1%, and given that these analyses rely on only 30 Asian participants, we do not advance strong interpretations of this finding here, though it points to the need to consider participant-level variation in future work on race categorization.

### Child racial categorization

Preliminary inspection of child response patterns revealed two participants who provided less than 50% usable data (i.e., less that 25 responses); they were excluded. In addition, subsequent to data collection two participants were identified as color blind, a factor that could affect how stimuli were perceived, especially for the skin color dimension. These two participants were also dropped (though preliminary analysis suggested a qualitatively similar pattern of data for these children). However, no substantive result reported here depends on any of these exclusions, which ultimately resulted in 71 usable participants (attrition = 5.3%). While the small number of non-White participants precluded exploring effects of participant race, to ensure that reported results were not importantly affected by this factor we conducted the primary analysis reported below with both the full sample and a reduced sample consisting only of White participants. In all cases the reduced sample yielded wholly consistent findings and we thus in what follows we focus on results from the full sample. In addition, no effects of participant gender appeared in the analyses described below.

#### Primary analyses

**Fig 5:**
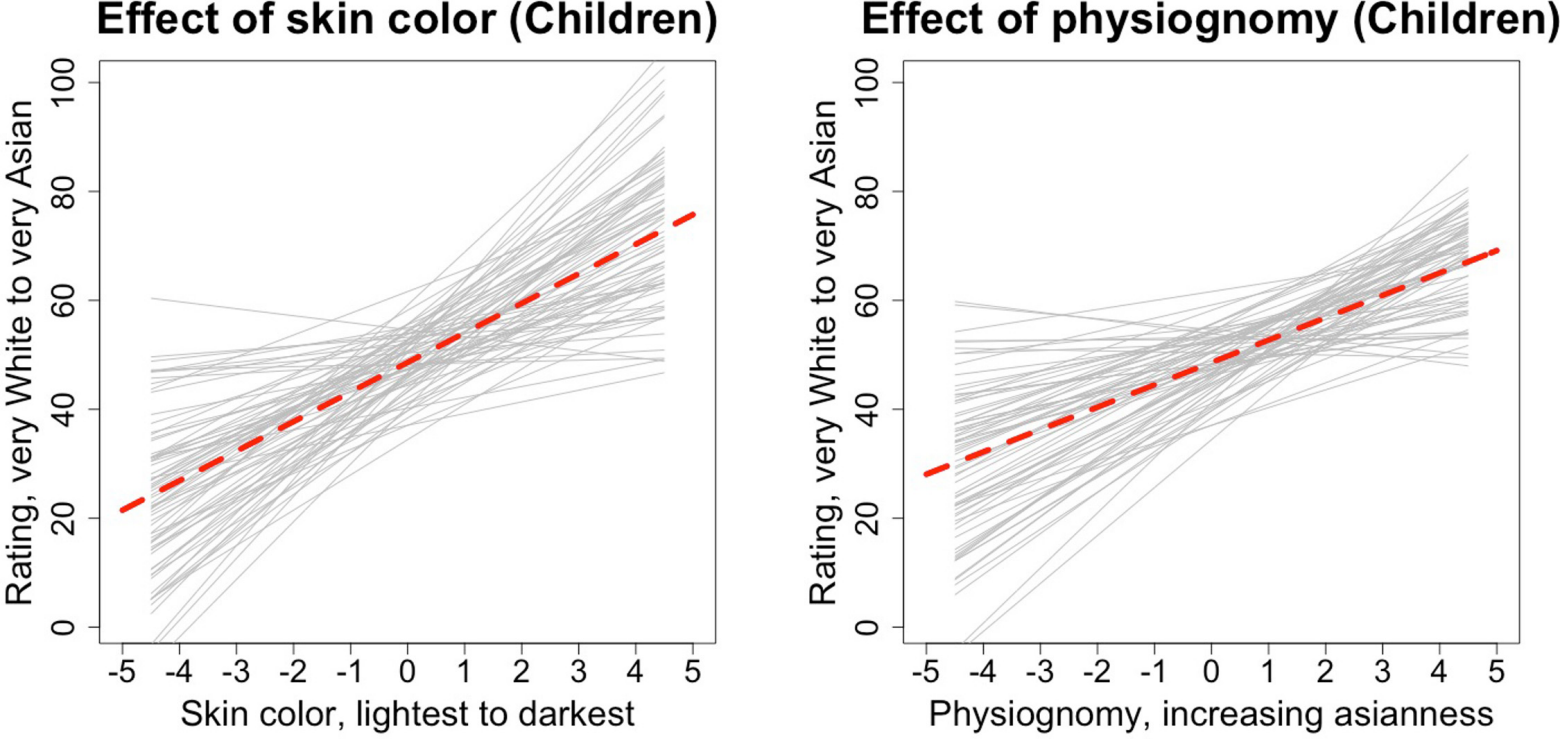
Main effects of (mean-centered) skin color and facial features when the other factor is set to its mean level, child participants only. Dashed red line represents overall model predictions and light grey lines represent the predictions for each participant, estimated from the random effects component of the models.

**Fig 6:**
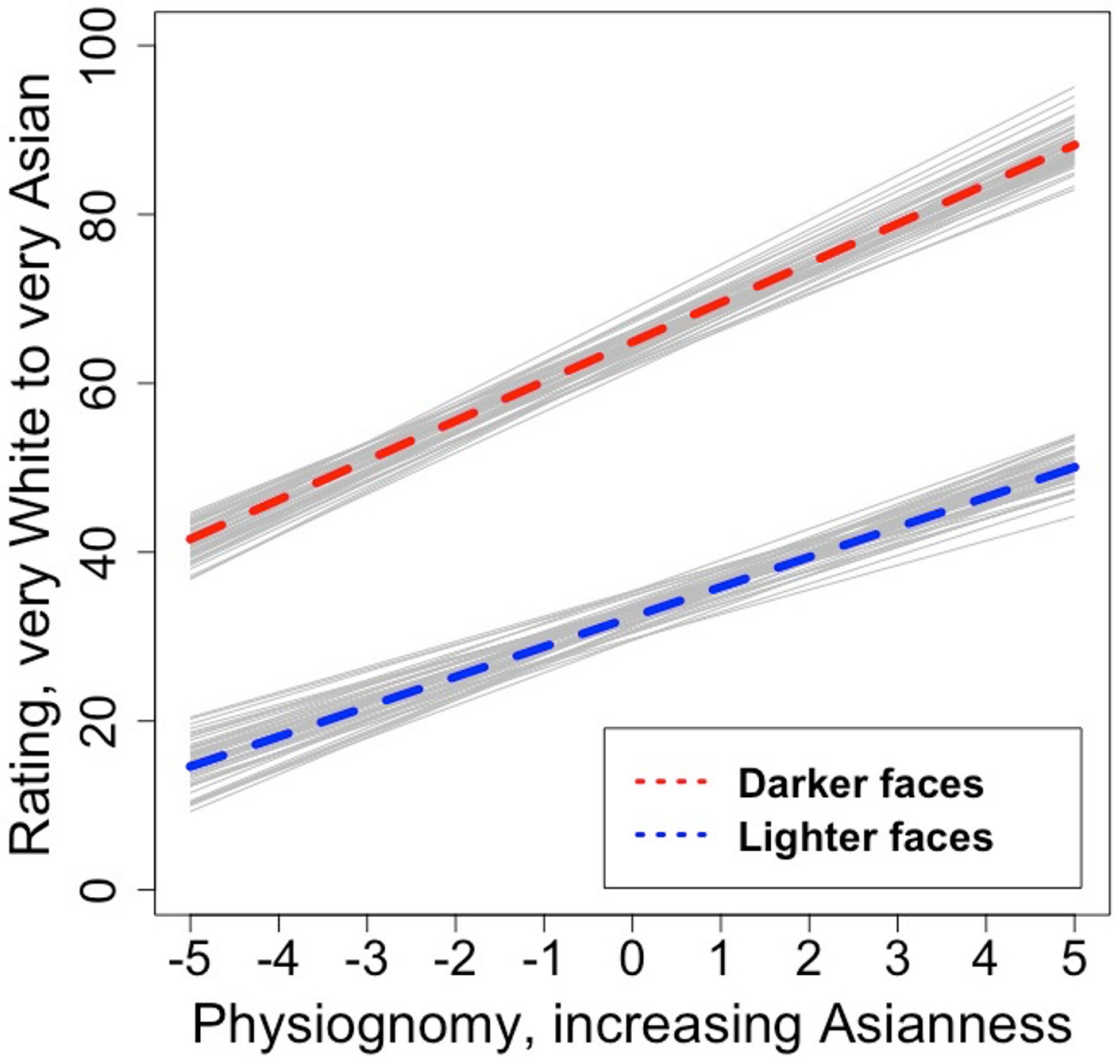
Interaction between facial features and skin color for children. Lighter and darker faces are depicted at the 3^rd^ and 8^th^ level of skin color, respectively. Light lines indicate uncertainty in the fitted regression.

A model incorporating our two main predictors and their interaction accounted for 32.9% of the trial-level variance as compared to an unconstrained model without predictors. In contrast with the approximately 70% variance explained in adults, this demonstrates greatly increased unexplained variance in children, most probably random or noisy responding, though it is worth acknowledging that we also have less data per stimulus with child participants, potentially leading to lower signal to noise. Broadly, however, results were similar to what we observed in adults. The intercept was again near the scale midpoint at 48.61 [*CI* = 46.87; 50.35], and there were also significant effects of all predictors: skin color: *B* = 4.42 [*CI* = 4.62; 6.23], physiognomy: *B* = 4.11 [*CI* = 3.42; 4.79], skin color x physiognomy interaction: *B* = 0.19 [*CI* = 0.08; 0.30]. Thus, as with adults, both skin color and physiognomy contributed. However, in this case the effect of skin color accounted for more residual variance (20.3%) as compared to physiognomy (12.4%). As with adults, the effect of physiognomy was more pronounced for faces more Asian in color and hue (though this effect was again quite small, accounting here for less than 0.5% explained variance). These main effects are depicted in Fig 5; Fig 6 depicts the effect of physiognomy at two 3^rd^ and 8^th^ level of skin color, representing estimated slopes of *B*_*skin = 3*_ *=* 3.63 [*CI* = 2.90; 4.36] and *B*_*skin = 3*_ *=* 4.58 [*CI* = 3.85; 5.31], respectively.

### Effects of Age on Racial Categorization

To examine effects of age we first compare adults to children considered as a group, and then examine potential age-related change within our child sample. To accomplish the former we included a dummy-coded predictor indicating age group, while the latter incorporated age as a continuous predictor for the child sample considered separately.

#### Comparing adult and child participants

Including age group (dummy coded as 1 for adult and 0 for child), skin color, physiognomy, and all interactions, but collapsing across stimulus set, explained 59.3% of the trial-level variance that was unexplained in an unconstrained model with no predictors. The three-way interaction between all three predictors did not improve model fitness, as evidenced by a *χ*^*2*^ goodness of fit test comparing the full model to a model dropping that interaction, *p* = .80. Thus, that term was dropped from the final model, leaving all three predictors and their two-way interactions. The effect of age group did not differ from 0, *B* = -.60 [*CI* = -2.55; 1.35], indicating that mean ratings did not differ across age groups, but this term was retained because it figured in higher-order interactions. Both the effects of skin color (*B* = 5.44 [*CI* = 4.82; 6.06]) and physiognomy (*B* = 4.10 [*CI* = 3.56; 4.63]) were significant, as was the skin color x physiognomy interaction (*B* = 0.20 [*CI* = 0.17; 0.24]). However, the two primary main effects interacted with age group, such that the effect of both skin color (*B* = 0.92 [*CI* = 0.14; 1.70]) and physiognomy (*B* = 1.95 [*CI* = 1.27; 2.62]) were stronger in adults than children, demonstrating age-related maturation in attention to both dimensions.

#### Age-related variation within the child sample

How does attention to these factors vary as a function of age if we focus solely on the child sample? We answer this question by modeling age as a continuous predictor, predicting categorization decisions as a function of (mean-centered) age, skin color, and physiognomy. Preliminary inspection, supported by a *χ*^*2*^ goodness of fit test, demonstrated that the 3-way interaction between all predictors did not add explanatory power (*p* = .13) and so that term was dropped. Substantively, this indicates that the interaction between skin color and physiognomy did not vary as a function of age.

In the resulting (final) model, the effect of age did not reliably differ from 0, demonstrating that mean ratings did not vary by age, *B* = .20 [*CI* = -.85; 1.25], but this term was retained because it figured in higher-order interactions. Both the effects of skin color (*B* = 5.42 [*CI* = 4.65; 6.20]) and physiognomy (*B* = 4.12 [*CI* = 3.52; 4.72]) were significant, as was the skin color x physiognomy interaction (*B* = 0.19 [*CI* = 0.08; 0.29]). However, the two primary main effects interacted with age, such that the effect of both skin color (*B* = 0.53 [*CI* = 0.07; 1.00]) and physiognomy (*B* = 0.84 [*CI* = 0.48; 1.20]) were stronger in older children, confirming that our age range captured substantial maturation in attention to the features that drive adult judgments.

### Subsidiary analyses to address missing stimuli

#### Effects of missing stimuli in the dataset

As noted in the methods section, above, a stimulus generation error led to the random omission of several stimuli and the corresponding duplication of neighboring stimuli (the full pattern of missing stimuli is presented in Fig 1). Consider each stimulus set a 10 x 10 array defined by 10 levels of skin color and 10 levels of physiognomy. If enough cells were missing, or if missing cells were over-represented in certain regions of the array, it could compromise model fitness, for example by limiting our ability to predict across the full range. It is important to note that no rows or columns were completely omitted; thus, participants were exposed to the full range of variation in both dimensions, though not the full linear combination of the two dimensions. Recall also that by design child participants received a random subset of stimuli, and so would not have received the full linear combination even if this stimulus issue had not occurred. Further, the mixed models employed here are generally robust against unbalance designs and missing data [23]. However, we were concerned with examining whether these stimuli omissions were problematic in terms of our models described above. Thus, we here detail several analyses aimed at exploring the potential impact of the missing stimuli. In brief, the examination strongly suggests that no such compromise is present, i.e. that the missing stimuli do not threaten the broader pattern of results we describe above.

We frame the question in the following way: Was the linear scaling of the predictors (i.e., of skin color and physiognomy) retained despite the presence of missing cells in the stimulus space? That is, despite the presence of missing cells, do responses track the actual values of skin color and physiognomy in the expected way? We focus on Stimulus Set 1, because it had the largest number of missing cells and because it was the only stimulus set used with children. We explore the question in two ways. First, we examine the correlations between the levels of the predictors and the average responses to stimuli at that level, which allows us to determine whether missing stimuli disrupts the expected pattern of covariation. Second, we examine the incremental step-sizes between observed responses to stimuli that were adjacent versus those that were not adjacent to a missing stimulus, on the logic that participants should respond based on the actual predictor values and so the observed step-sizes between stimuli with a gap between them should be approximately twice that of the observed step-sizes of adjacent stimuli without a gap. If this did not occur, it would suggest that there was some distortion in the scaling across the stimulus space.

First, for the correlational analysis we computed individual correlations between each row of the stimulus set (here defined as the 10 levels of skin color) and the mean observed value for every position in that row (with missing cells omitted). We then repeated this analysis for each column (there instead correlating the mean response with the corresponding level of physiognomy). This produced 10 row and 10 column correlations. The correlations were quite high in all cases, mean *r* = .96 (*SD* = .02, range: .93; .99.). In addition, the average correlation did not appear to vary as a function of the number of missing cells in that row or column. Rows and columns ranged from 0 to 2 missing values, and the average correlations for 0, 1, and 2 missing values were .97, .96, and .96, respectively. Thus, despite some missing cells, average ratings tracked very closely with actual values.

A second analysis tackled the question via step-size differences instead of correlations. Stimuli that are neighbors in row or column have a one-step difference between them in a given dimension, but where a cell is missing there is a two-step difference between a stimulus and the next available stimulus along that dimension. Our question is whether the average shift in response between cells with a gap between them is approximately twice the average shift in response between cells that are adjacent, as we would predict if participants are responsive to the full range of variation even though some cells are missing. This question cannot be examined via the linear models described above because they impose linearity on the data; however, the *observed* mean values for each stimulus do allow us to examine this question. We therefore computed the step-sizes between stimuli (in both rows and columns), calculating the average difference between directly adjacent stimuli (where there were no missing stimuli) and the average difference between stimuli that are two steps apart (because of a missing cell between them). Because we did not want to make the assumption that skin color and physiognomy had equal step sizes, we did this separately for rows (physiognomy) and columns (skin color). In both cases, the evidence supported the expected linear scaling, even where cells were missing. Starting with skin color, the average step-size for adjacent cells was 5.6 [95% *CI* = 4.46; 6.83]; i.e., moving one column to the right corresponded to a 5.6 point shift towards the Asian end of the scale. For cells with an intervening gap due to a missing cell, the average was approximately twice as large, *M* = 10.6 [5.51; 15.69]. Turning to physiognomy, the pattern of results was much the same. The average step-size for adjacent cells was 4.2 [2.89; 5.57], and the average step-size over a missing cell was 10.4 [5.47; 15.33]. Thus, in both cases the observed step sizes suggest that participants were quite sensitive to the differences between stimuli despite the presence of missing cells.

#### Adult replication with novel stimulus set

To further ensure that our results were not compromised by missing stimuli we ran another version of the adult study using a novel 10 x 10 grid of faces and sought to replicate the results reported above. An initial model predicting racial categorization as a function of skin color, physiognomy, and stimulus set accounted for 61.4% of the trial-level variance that was unexplained in the unconstrained model. The effects of skin color, physiognomy, and the interaction between them were all significant and of quite similar magnitudes to the effects of the three stimulus sets in the main. The intercept, here representing the mean rating across all stimuli, was 43.17 [*CI* = 41.66; 44.67]. There were significant effects of both skin color, *B* = 6.89 [*CI* = 6.75; 7.03] and physiognomy, *B* = 5.74 [*CI* = 5.59; 5.89], as well as an interaction between them, such that the effect of physiognomy was stronger when skin was darker, *B* = 0.31 [*CI* = .26; .36]. As can be seen in the final row of Table 1 (above), these estimates paint a very similar picture to the results of the main study and suggest that missing stimuli did not have a strong effect on the pattern of results described above.

## Discussion

We investigated the developmental emergence of adult-like perceptual categorization for the Asian-White category boundary using a continuous measure and a large set of stimuli that systematically varied with respect to skin color and other aspects of facial physiognomy. Beginning with adults, our primary finding is that adults balanced attention to skin color versus other physiognomic cues, with similar amounts of variance explained by each. Turning to children, in the very broadest of strokes children showed a similar pattern, evincing sensitivity to both factors across the age ranges tested here.

However, children’s responses were considerably less systematic than those of adults, with our predictors jointly accounting for only about half the variance accounted for by adult judgments. We interpret this as showing that the White-Asian distinction is perceptually challenging, necessitating a longer road to adult-like performance. Further, our data suggest that the difficulty is centered on the particular challenge of the physiognomic cues rather than the skin color cues. While physiognomy and skin color had approximately equal impact for adults, physiognomy was a less powerful predictor than skin color across the child sample, suggesting that even in our oldest children it did not yet approach its mature form.

Another interesting aspect of the present findings is the interaction between skin color and physiognomy. For both children and adults (and especially for Asian adults) the influence of one factor increased as the other factor became more pronounced (i.e., there was a positively signed interaction between them). Such a pattern could be explained by associative links between skin color and physiognomy such that they mutually reinforce one another. A second possibility is that skin color contrasts influence the perception of features more directly. This could come about because the skin color dimension in our stimuli is a complex pattern created by the independent action of 50 texture dimensions; if some of those textural dimensions enhance contrast around features that are diagnostic of category membership they could produce the positive interaction revealed here. It is difficult to rule this possibility in or out at present, but two points are worth making. First, even if this pattern holds, it would remain an open question whether that is a feature of our particular stimulus set or a genuine feature of the Asian-White perceptual contrast in the real world. Second, the interaction, while statistically significant, was a weak effect, accounting for less than 1% of modeled variance. Thus, while intriguing it does not have a major impact on the interpretation of our results.

Because our past study of the White-Black category boundary used an identical method [14], it is fruitful to compare the general pattern of results (thought we have elected not to do directly quantitative comparisons due to some differences in sample composition). Perhaps the most striking difference concerns the relative strength of the skin color and physiognomic dimensions in influencing categorization judgments. As measured by the proportional variance explained, adults were similarly consistent in the two categorization tasks. But while skin color and physiognomy were roughly equal contributors to White-Asian judgments, skin color was approximately four times as powerful a predictor of adult White-Black judgments. This likely reflects genuine differences in the features that are most diagnostic of the category boundary, validating the intuition that skin color is a less clear cue to the White-Asian than the White-Black category boundary. Indeed, young children showed a near-total reliance on skin color in the case of the Black-White racial distinction, but did reliably attend to physiognomic cues in the White-Asian case (even if they were a less powerful predictor than skin color cues). We are aware of no reason to think that the physiognomic contrasts themselves are more difficult in one case than another and therefore suggest that the inattention to physiognomic cues in the case of the White-Black contrast is not due to an inability to track or encode those cues per se. Rather, it now seems more plausible that, because skin color is such a perceptually powerful guide to the White-Black distinction, children adopt it as their initial heuristic, only beginning to attend to other features as they develop increased perceptual expertise and/or begin to note the occasional mismatches between their own and adult judgments. By contrast, because skin color alone is a less reliable heuristic for the Asian-White boundary, children begin to shift more attentional resources to physiognomic cues from earlier in development. The implication, then, is that race perception has particularities that are directly inherited from the specific category distinction in question. Future work could strengthen this argument by focusing on children’s ability to draw on the specific feature cues that mark each category boundary [26], or by widening the inquiry to other racial category boundaries.

Another notable difference between the White-Black and White-Asian case concerns the interaction between skin color and other facial features. While an interaction was reported in both cases, the direction of this interaction differed across the two contrasts. For the White-Black contrast the interaction was negative, demonstrating that as one cue became weaker, the other cue exerted a larger influence. This has been interpreted as evidence for a desire to enforce the category boundary, a form of ingroup overexclusion [14,27]. But given that ingroup overexclusion would seem to be a viable possibility for White participants for both ambiguously Black and Asian faces, this interpretation cannot account for the opposite pattern across the two cases. Given that in both cases the interactions were relatively weak, we do not want to place too much weight on these divergent findings, but it would be useful for future work to confirm the general interactive patterns.

While these results should be interpreted cautiously given the small samples involved, they are also suggestive of differences in race categorization between White and Asian adult participants. More specifically, White adults rated faces as somewhat more Asian on average, a finding consistent with hypodescent, i.e. the tendency to assign ambiguous stimuli to a lower-status racial outgroup [28], as well as the more general phenomenon of “ingroup over-exclusion” in which ambiguous stimuli are more likely to be excluded from the ingroup [27]. Further, the interaction described in the preceding paragraph, in which skin color and other aspects of physiognomy had a super-additive effect on race categorization, was stronger for Asian participants, suggesting that these two types of cues are more holistically linked in Asian’s perceptual model of race. It is also important to acknowledge that our work is limited by the relatively small number of non-White participants in our child sample. It would be particularly interesting for future work to explore how apparent differences in White and Asian adults emerge over developmental time, or how the emergence of the White-Asian perceptual distinction unfolds in mixed race children, children who are neither White nor Asian, or Asian children who were not raised in the US. Future work including these broader range of samples is critical to grappling with the question of how social contexts shape racial category distinctions.

Relatedly, it seems highly likely that individual differences in experience, such as interracial contact opportunities, will affect the development of racial category boundaries. While our sample likely contained at least some variation along this dimension, we unfortunately did not include measures that would allow us to examine this issue. Future work could address this either by more focused sampling to include variation in contact experience (e.g. by comparing more and less diverse schools) or by including measures of interracial contact experience.

One aspect of our investigation that is worthy of note is our use of three distinct stimulus sets in the main study. These stimulus sets did not produce identical results (see Table 1). In particular, the strength of the interaction between skin color and physiognomy varied somewhat across sets, and was notably lower in one of the sets (Set 3). Each grid was created by interpolating between two base faces, one Asian and one White; different base faces thus produce different grids because of the differences between base faces. While all base faces were considered highly prototypical by both FaceGen and our participants (for whom the base faces represented the faces at the diagonal of White or Asian in both dimensions), they varied in unique ways based on adding random noise to the 100 FaceGen dimensions. No one grid should be considered definitive, but the fact that including a predictor for which grid was employed only accounted for about 1% of modeled variance, and the fact that all model estimates were similar in magnitude and identical in direction, gives us confidence that as a whole we are successfully modeling the perceptual dynamics of the category boundary and that they do not depend on a single pair of base faces.

Our use of a continuous measure rather than a dichotomous judgment had the advantage of providing a fine-grained picture of how faces are judged, but it would also be useful for future work to examine dichotomous race judgments. After all, in many contexts, the actual use of racial categories has this more dichotomous category, and the function by which continuous judgments relate to categorical judgments is not obvious. Further, future work could explore photographic rather than computer-generated faces. While computer-generated faces allow finer control and systematic variation of features, and usually approximate results from studies employing photographs, there are also cases in which results differ based on stimulus modality [29–31]. Thus, confirming the primary findings of the present study with real faces would be useful. In addition, future work could expand on this pattern in several ways, most notably by incorporating other types of stimuli. For example, the present research focused solely on male faces, raising the question of whether female faces would pattern similarly. This question is important because of other evidence of interactive patterns between race and gender perception [32], but answering it will require further work. In addition, incorporating a wider range of racial category variation in the same study would be useful, for example by including White, Black, East Asian, and South Asian faces; South Asian faces might be particularly interesting in that at least intuitively they feature a salient distinction in skin color but a relatively weaker distinction in terms of other physiognomic features.

Our work also makes contact with a growing body of research on the perception and memory of biracial or multiracial faces [33]. This work has provided evidence of hypodescent [34] and has also suggested that categorizing faces *as* biracial is cognitively more taxing for many individuals [35]. Further, memory for biracial faces has shown to be affected by the label used to refer to the face at encoding [36] and to be biased at recall towards the category it resembles most closely [37]. While our task did not explicitly engage with biraciality it did involve categorizing a large number of faces that could be described as biracial, given that they were drawn from a perceptual space intermediate between White and Asian. Interestingly, in our task participant ratings did not show clear evidence of hypodescent or other category boundary accentuation effects [38], as evidenced by a mean rating across all trials quite near the middle of the scale (i.e., near 50). One possibility is that such effects, even if they initially occur, disappear as participants rapidly calibrate to a long sequence of highly variable faces. Nonetheless, the present methodology could fruitfully be extended to the study of biracial categorization, for example asking participants to categorize target faces as White, Asian, or biracial.

In closing, the study of race categorization is interesting as an inquiry into a basic yet challenging aspect of perceptual learning, but also as a component of other aspects of intergroup social cognition such as stereotyping and prejudice, which presuppose the partitioning of individuals into social categories. Studying the acquisition of adult-like perceptual categories is therefore an interesting window into developmental social cognition.
